# Species identification and comparative population genetics of four coastal houndsharks based on novel NGS‐mined microsatellites

**DOI:** 10.1002/ece3.2770

**Published:** 2017-02-05

**Authors:** Simo N. Maduna, Charné Rossouw, Charlene da Silva, Michelle Soekoe, Aletta E. Bester‐van der Merwe

**Affiliations:** ^1^ Department of GeneticsMolecular Breeding and Biodiversity GroupStellenbosch UniversityStellenboschSouth Africa; ^2^ Department of Agriculture, Forestry and FisheriesFisheries ResearchRogge BaySouth Africa; ^3^ Department of Ichthyology and Fisheries ScienceRhodes UniversityGrahamstownSouth Africa

**Keywords:** cross‐amplification, *Galeorhinus galeus*, Illumina sequencing, microsatellites, *Mustelus mustelus*, *Mustelus palumbes*, *Triakis megalopterus*

## Abstract

The common smooth‐hound (*Mustelus mustelus*) is the topmost bio‐economically and recreationally important shark species in southern Africa, western Africa, and Mediterranean Sea. Here, we used the Illumina HiSeq™ 2000 next‐generation sequencing (NGS) technology to develop novel microsatellite markers for *Mustelus mustelus*. Two microsatellite multiplex panels were constructed from 11 polymorphic loci and characterized in two populations of *Mustelus mustelus* representative of its South African distribution. The markers were then tested for cross‐species utility in *Galeorhinus galeus*,* Mustelus palumbes*, and *Triakis megalopterus*, three other demersal coastal sharks also subjected to recreational and/or commercial fishery pressures in South Africa. We assessed genetic diversity (*N*_A_, *A*_R_, *H*_O_, *H*_E,_ and PIC) and differentiation (*F*_ST_ and *D*_est_) for each species and also examined the potential use of these markers in species assignment. In each of the four species, all 11 microsatellites were variable with up to a mean *N*_A_ of 8, *A*_R_ up to 7.5, *H*_E_ and PIC as high as 0.842. We were able to reject genetic homogeneity for all species investigated here except for *T*. *megalopterus*. We found that the panel of the microsatellite markers developed in this study could discriminate between the study species, particularly for those that are morphologically very similar. Our study provides molecular tools to address ecological and evolutionary questions vital to the conservation and management of these locally and globally exploited shark species.

## 1 Introduction

Sharks play a crucial role in maintaining the ecological balance in marine ecosystems as keystone species, yet these animals are gradually declining worldwide in seascapes heavily impacted by humans (Dulvy et al., 2014). Such declines in wild populations not only will have negative ecological impacts on lower trophic species (Price, O'Bryhim, Jones, & Lance, 2015) but can also alter the levels and distribution of genetic diversity among populations (Dudgeon et al., 2012). It is likely that sharks may not respond well to population declines compared to other marine fishes owing to their *K*‐selected life‐history traits, *i*.*e*., slow growth, late maturity, and low reproductive outputs (Compagno, 1984; Ebert, Fowler, Compagno, & Dando, 2013). This highlights the need for conservation and management measures to ensure the sustainable utilization of these fishery resources. Implementing such measures often requires information on fishery dynamics, biological and baseline ecological data which in most cases are not yet available (Velez‐Zuazo, Alfaro‐Shigueto, Mangel, Papa, & Agnarsson, 2015). Molecular approaches have been very useful in providing insight into historical and contemporary demography of various commercially important shark species, especially with respect to population connectivity, stock structure, and metapopulation dynamics (Boomer, 2013; Chabot, Espinoza, Mascareñas‐Osorio, & Rocha‐Olivares, 2015; Pereyra, García, Miller, Oviedo, & Domingo, 2010; Sandoval‐Castillo & Beheregaray, 2015).

Despite ongoing sampling difficulties, population genetics studies of bio‐economically important sharks are now fast increasing due to molecular genetic markers becoming more readily available. For example, next‐generation sequencing (NGS) has become a common approach to developing microsatellites in nonmodel organisms as it enables the recovery of thousands of repeat‐containing sequences at a reduced time and cost (Blower, Corley, Hereward, Riginos, & Ovenden, 2015; Chabot & Nigenda, 2011; Pirog, Blaison, Jaquemet, Soria, & Magalon, 2015). Also, newly developed microsatellites for source species can be assessed for cross‐species transferability in congeneric and confamilial (target) species and have shown to have a high success rate in elasmobranchs (Blower et al., 2015; Boomer & Stow, 2010; Chabot, 2012; Maduna, Rossouw, Roodt‐wilding, & Bester‐van der Merwe, 2014; Pirog et al., 2015). This allows for the development of a standardized panel of microsatellite multiplex PCRs for comparative population genetics studies and identification of species.

Identification of bio‐economically important sharks during port inspections is very difficult (or even impossible) when using traditional taxonomic tools because of carcass processing at sea, where the head and fins are removed (Abercrombie, Clarke, & Shivji, 2005; Akhilesh et al., 2014; Stevens, 2004). During processing morphological and meristic criteria which are pivotal to the accurate identification of specimens are lost (Mendonça et al., 2010; da Silva & Bürgener, 2007). Several different genetic identification methods have previously been developed to resolve misidentification issues (Blanco, Pérez‐Martín, & Sotelo, 2008; Naylor et al., 2012; Ward, Holmes, White, & Last, 2008) or to reveal captures of threatened shark species (Clarke et al., 2006; Liu, Chan, Lin, Hu, & Chen, 2013; Shivji, Chapman, Pikitch, & Raymond, 2005). These include gel‐based identification methods (Farrell, Clarke, & Mariani, 2009; Pank, Stanhope, Natanson, Kohler, & Shivji, 2001), DNA barcoding (using the cytochrome oxidase *c* subunit I; Ward et al., 2008), sequenced‐based identification method (using sequences of the cytochrome *b*; (Blanco et al., 2008) or NADH dehydrogenase subunit 2 gene regions (Naylor et al., 2012)), and high‐resolution melting analysis (Morgan et al., 2011). Furthermore, a few studies have recently demonstrated the applicability of cross‐species microsatellites for species identification based on species‐specific allele sizes (Marino et al., 2014) and distinctive allele frequencies at multiple loci (Giresi et al., 2015; Maduna et al., 2014).

South Africa is an ecologically and evolutionarily dynamic region with a diverse elasmobranch fauna (Bester‐van der Merwe & Gledhill, 2015; Compagno, 1984; Ebert et al., 2013) and is located in the transition zone between the Atlantic and Indo‐Pacific biomes (Briggs & Bowen, 2012). The Atlantic/Indian Ocean boundary in this region is characterized by two ocean basins, the Southeast Atlantic Ocean (SEAO) and Southwest Indian Ocean (SWIO) with two major currents, the cold Benguela Current and the warm Agulhas Current (Briggs & Bowen, 2012; Hutchings et al., 2009). Thus far, only a few regional population genetics studies related to sharks have been conducted in southern Africa but have shed some light on the possible impact of oceanographic features on gene flow patterns of species affected by fisheries, including the tope shark (*Galeorhinus galeus*), common smooth‐hound (*Mustelus mustelus*), and spotted gully shark (*Triakis megalopterus*) (Bitalo, Maduna, da Silva, Roodt‐Wilding, & Bester‐van der Merwe, 2015; Maduna, da Silva, Wintner, Roodt‐Wilding, & Bester‐van der Merwe, 2016; Soekoe, 2016). These studies showed that the interaction between the two ocean currents plays a prominent role in limiting dispersal around the southern tip of Africa, particularly in an eastward direction for the common smooth‐hound shark for example. Given that single‐species conservation strategies do not adequately protect the biological and ecological needs of multiple species within threatened ecosystems, the focus has shifted toward multispecies approaches.

**Figure 1:**
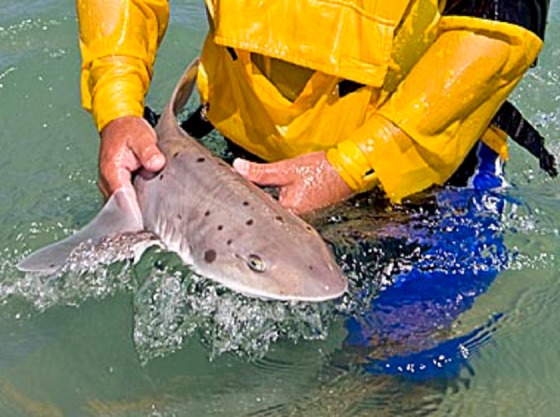
*Mustelus mustelus*. An individual of *M*. *msutelus* with evident black spots on the dorsal surface. Picture by Rob Tarr

The local distribution ranges of all the triakid species (family Triakidae) investigated here, the tope shark, common smooth‐hound, whitespotted smooth‐hound (*M*. *palumbes*), and the spotted gully shark, extend across the Atlantic/Indian Ocean boundary. This presents an ideal opportunity to test whether the interplay of oceanographic features and life‐history traits are the drivers of population subdivision in these sharks. The tope shark is a highly mobile semipelagic demersal species that is widely distributed in temperate waters (Ebert et al., 2013). Although sexual maturity depends on the ocean basin of origins, females reach sexual maturity at a total length (*L*_T_) of 118–150 cm and males at 107–135 cm *L*_T_. Reproduction is viviparous (no yolk‐sac placenta) with a triennial reproductive cycle (Ebert et al., 2013; Lucifora, Menni, & Escalante, 2004; McCord, 2005). Conversely, smooth‐hounds are relatively small and less mobile epibenthic sharks (<170 cm *L*_T_) (da Silva et al., 2013; Smale & Compagno, 1997). The common smooth‐hound (Figure 1) is a cosmopolitan species distributed across the Mediterranean Sea, the eastern Atlantic Ocean, and the Southwest Indian Ocean, whereas the whitespotted smooth‐hound is endemic to southern Africa and is found from Namibia to northern KwaZulu‐Natal (Ebert et al., 2013; Smale & Compagno, 1997). Reproduction in the common smooth‐hound is characterized by placental viviparity and a seasonal reproductive cycle whereby each cycle may take 1 year or longer. Sexual maturity is reached at 70–112 cm *L*_T_ for males and 107.5–124 cm *L*_T_ for females (Saïdi, Bradaï, & Bouaïn, 2008; Smale & Compagno, 1997). For the whitespotted smooth‐hound, reproduction is characterized by aplacental viviparity and an aseasonal reproductive cycle although the timing of reproductive cycles is presently unclear. Sexual maturity is reached at 75–85 cm *L*_T_ for males and 80–100 cm *L*_T_ for females (Ebert et al., 2013; Smale & Compagno, 1997). Similar to smooth‐hounds morphologically but with a larger body size, the spotted gully shark is endemic to southern Africa and is found from southern Angola to Coffee Bay, South Africa. Reproduction is ovoviviparous with a biennial to triennial reproductive cycle (Smale & Goosen, 1999; Soekoe, 2016). Sexual maturity is reached at 94–130 cm *L*_T_ for males and 140–150 cm *L*_T_ for females. Anecdotal evidence based on tagging data suggests that the spotted gully sharks exhibit a high degree of site fidelity or residency because *ca*. 80% of these animals were recaptured close to their release site (within a 20‐km radius), regardless of the time at liberty (Dunlop & Mann, 2014; Soekoe, 2016).

Here we characterize a set of NGS‐mined microsatellites in common smooth‐hound and evaluate the potential of cross‐species utility of these markers in species identification and assessing the distribution of genetic variation across populations sampled along the South African coast.

## 2 Materials and Methods

### 2.1 Sample collection and genomic DNA extraction

**Figure 2:**
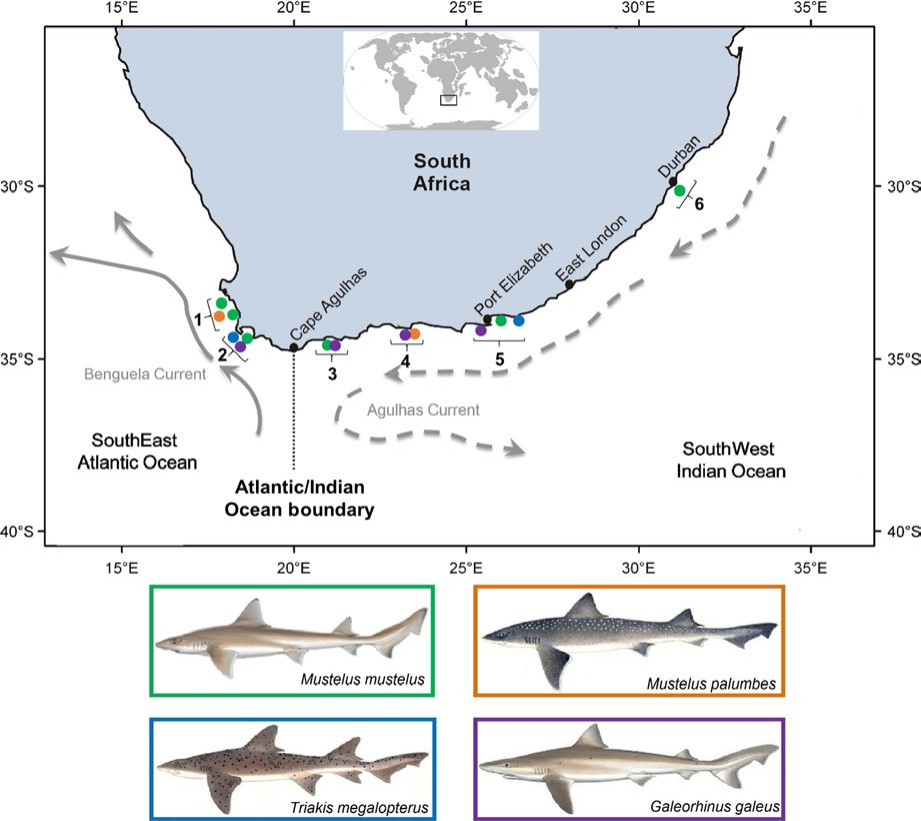
Sampling localities of four coastal shark species with the green circle representing *Mustelus mustelus*, and orange, blue, and purple circles representing *Mustelus palumbes*,* Triakis megalopterus,* and *Galeorhinus galeus*, respectively. Locations 1–2 and 3–6 represent the South African Southeast Atlantic and Southwest Indian Ocean sampled populations, respectively. The major oceanographic features are also shown

A total of 144 finclip samples from four coastal shark species (the tope shark, common smooth‐hound, whitespotted smooth‐hound, and the spotted gully shark) were examined (Table 1). We included samples from the west and east coasts, representing the two main ocean basins (SEAO and SWIO) spanning the South African coastline (Figure 2). The west coast samples represent SEAO individuals collected west of the Atlantic/Indian Ocean boundary, while the east coast samples represent SWIO individuals collected east of the Atlantic/Indian boundary. In addition, we obtained tissues samples from three individuals each of the starry smooth‐hound (*Mustelus asterias*) and the blackspotted smooth‐hound (*M. punctulatus*) from the Mediterranean Sea, and two individuals of the hardnose smooth‐hound (*M*. *mosis*) from Oman in the northwestern Indian Ocean. Total genomic DNA was isolated using a standard cetyltrimethylammonium bromide (CTAB) extraction protocol of Sambrook and Russell (2001). The concentration and the quality of the extracted DNA were determined by measuring its optical density at 260 nm (A_260_) and 280 nm (A_280_) with a NanoDrop ND 2000 spectrophotometer (Thermo Fisher Scientific; www.thermofisher.com). A small subset of samples was subjected to electrophoresis in 1× TAE buffer for 1 hr at 80 V. Five microliters of the isolated genomic DNA was loaded on 0.8% agarose gel stained with ethidium bromide to check DNA quality. The gels were photographed under a Gel Documentation system (Gel Doc XR+, Bio‐Rad, South Africa).

**Table 1:** Details of the sampling locations and sample sizes (*N*) of four coastal shark species

Species	Ocean basin	Collection site	Geographic coordinates	*N*
*Mustelus mustelus* (*N *=* *48)	SEAO	Langebaan Lagoon	33°09′S, 18°04′E	8
		Robben Island	33°48′S, 18°24′E	8
		False Bay	34°10′S, 18°36′E	8
	SWIO	Struis Bay	34°47′S, 20°03′E	8
		Jeffreys Bay	34°35′S, 24°56′E	8
		Durban	29°44′S, 31°07′E	8
*Mustelus palumbes* (*N *=* *40)	SEAO	Yzerfontein	33°20′S, 18°02′E	11
	SWIO	Mossel Bay	34°09′S, 22°10′E	13
	Unknown	–	–	16
*Galeorhinus galeus* (*N *=* *24)	SEAO	Robben Island	33°48′S, 18°24′E	7
		False Bay	34°10′S, 18°36′E	7
	SWIO	Struis Bay	34°47′S, 20°03′E	3
		Mossel Bay	34°09′S, 22°10′E	2
		Port Elizabeth	34°04′S, 25°03′E	5
*Triakis megalopterus* (*N *=* *32)	SEAO	Cape Point	34°20′S, 18°33′E	8
		Betty's Bay	34°22′S, 18°55′E	8
	SWIO	Port Elizabeth	34°04′S, 25°03′E	16

### 2.2 Development of species‐specific microsatellites

Total genomic DNA from one individual of common smooth‐hound was isolated and sent to the Agricultural Research Council Biotechnology Platform in Pretoria, South Africa. One microgram of genomic DNA was used for 2 × 250 bp paired‐end library preparation with a mean insert size of 400 bp using the standard Nextera™ library preparation kit (Illumina). The library was sequenced on two lanes of an Illumina HiSeq™ 2000 sequencer. The generated sequence reads were submitted to a quality control (QC) step to remove artificial duplicates and/or reads that contained any “Ns” using PRINSEQ 0.20.4 (Schmieder & Edwards, 2011). Reads were quality‐filtered and trimmed to remove all Nextera adapters and sequences shorter than 35 bp using TRIMMOMATIC v. 0.33 (Bolger, Lohse, & Usadel, 2014) with default settings. We selected a Phred quality score of 15 and filtered for sequences that contained at least 90% of the individual bases above this quality score. To check whether primer, barcode, and adapter sequences have been properly trimmed, we visualized the sequencing quality using the software FASTQC v. 0.11.4 (Andrews, 2010). After the QC step, we built contigs from read files using ABYSS v. 1.5.2 (Simpson et al., 2009) and selected contigs larger than 250 bp for microsatellite identification in MISA v. 1.0 (Thiel, Michalek, Varshney, & Graner, 2003). Sequences with ≥5 uninterrupted motifs toward the middle were selected and blasted against the NCBI database to filter for the contigs which contained hits with microsatellites against other elasmobranch or teleost species. Sequences with hits were selected for primer design using PRIMER3 v. 0.4.0 (Untergrasser et al., 2012).

### 2.3 Microsatellite validation, cross‐species amplification, and species identification

Polymerase chain reaction (PCR) was carried out on a GeneAmp® PCR System 2700 in a 10 μL reaction volume that included 50 ng of template DNA, 1× PCR buffer, 200 μmol/L of each dNTP, 0.2 μmol/L of each primer, 1.5 mmol/L MgCl_2_, and 0.1 U of GoTaq® DNA polymerase. The PCR cycling conditions were as follows: (1) one cycles of initial denaturation at 95°C for 2 min, (2) 35 cycles of denaturation at 94°C for 30 s, optimized annealing temperature (*T*_A_) for 30 s, elongation at 72°C for 2 min, (3) a final elongation of one cycle at 60°C for 5 min and thereafter stored at 4°C. Optimum annealing temperature was determined by experimental standardization for each of the primer pairs (Table 2). Amplification products were subjected to agarose gel electrophoresis to determine their size. Levels of polymorphism were initially assessed at all the successfully amplified microsatellite loci in a panel of eight individuals of *M*. *mustelus*. The amplified PCR products were resolved on a vertical nondenaturing 12% polyacrylamide gel to detect size variants. We considered microsatellites to be polymorphic when two bands were distinguishable in a single individual (i.e., heterozygote), and/or we observed clear size differences between different individuals. Polymorphic microsatellite loci were selected and primers fluorescently labeled with one of the following dyes: FAM, VIC, PET, or NED followed by multiplex optimization of two mutiplex assays (MPS1 and MPS2). A panel of 48 individual *M*. *mustelus* representatives of the two ocean basins (SEAO and SWIO) was genotyped for marker characterization. Multiplex PCR conditions were realized using the Qiagen Multiplex PCR kit and conducted according to the manufacturer's instructions except for varying primer concentrations (Table 3) and *T*_A_, 56°C for MPS1 and 57°C for MPS2. For subsequent analysis on an ABI 3730XL DNA Analyzer, PCR products were diluted in distilled water and fragment analysis performed together with the LIZ600 internal size standard. Individual genotypes were scored based on fragment size via GENEMAPPER v. 4.0 (Life Technologies, South Africa). To determine the utility of these markers for future regional studies of intra‐ and interspecific genetic diversity in houndsharks (Triakidae), we also tested the 11 microsatellite loci on the blackspotted smooth‐hound, spotted gully shark, starry smooth‐hound, tope shark, and whitespotted smooth‐hound using the PCRs and microsatellite genotyping conditions described previously.

**Table 2:** Details of 15 microsatellite loci developed for *Mustelus mustelus*

Locus name	Contig ID	Primer sequence	Motif	PCR product size	*T*_A_ (°C)	BLAST hit	PAGE results	Accession no.
Mmu1	603691 889 5405	F‐CCCCATTTGCAAACAGAGTT	(AT)_7_	210	57	*Danio rerio*	Polymorphic	KX261856
		R‐ATTTCCCGCTGTTACATTGC						
Mmu2	17731961 828 2935	F‐TTGTCTGCAGGAAACACAGC	(AC)_6_	163	56	*Cyprinus carpio *	Polymorphic	KX261857
		R‐GCATCGTGTGAAATGGGAAT						
Mmu3	19222092 770 1754	F‐ATACACGGACCGACTCGAAC	(TC)_7_	240	56	*Astyanax mexicanus*	Polymorphic	KX261858
		R‐TAATGCCGAGATCAGGAACC						
Mmu4	26403340 740 2107	F‐TCCATCCAGCGTTAAAGGAC	(TG)_7_	173	56	*Astyanax mexicanus*	Polymorphic	KX261859
		R‐GCACCAGAGCTTCCCATTTA						
Mmu5	12961358 707 1755	F‐ACCACTCCCTGCAGCACTAC	(CTC)_6_	282	57	*Callorhinchus milii*	Polymorphic	KX261860
		R‐AGGAGATGCTTTGGCACTTG						
Mmu6	14682365 1857 7998	F‐CACCGGAGACCTCTAACTGG	(CGC)_6_	212	57	*Chrysemys picta bellii *	Polymorphic	KX261861
		R‐CGATGATGATGAAGGACGTG						
Mmu7	18961995 1089 3100	F‐TCCCTCATTTGCTTCAGGAG	(GCT)_5_	219	57	*Callorhinchus milii *	Polymorphic	KX261862
		R‐CGACATGAAACGCAGAAAGA						
Mmu8	26836431 1069 3092	F‐AGTAAGGCGCGCTATGATTG	(CAG)_5_(TGT)_5_	431	56	*Callorhinchus milii*	Polymorphic	KX261863
		R‐TAGAAGTCATCGCCCTCCAC						
Mmu9	7951092 402 890	F‐ACGGTTCTGAGCAATCGTCT	(GAAT)_5_	172	56	*Callorhinchus milii *	Monomorphic	KX261864
		R‐TGCGATATTCGTCAGGTGAA						
Mmu10	11748443 647 1363	F‐AATCCTGAGCACCAGGACAC	(CATA)_5_	299	56	*Squalus acanthias*	Monomorphic	KX261865
		R‐TGTGTGAATTCCCCAGATGA						
Mmu11	61216 525 1150	F‐ATCTTGTTAACCGCCGACAG	(CAA)_5_	211	56	*Callorhinchus milii*	Polymorphic	KX261866
		R‐CGCCATGTTGATCGAAGTAA						
Mmu12	1178354	F‐GAGCAGCCAAGCATTAGTCC	(GAT)_6_	208	56	*Callorhinchus milii*	Monomorphic	KX261867
		R‐CGGCTTCAGAAATTGGAATC						
Mmu13	14447036	F‐TCATTCCTCACACCCACTCA	(GCA)_5_	112	56	*Squalus acanthias*	Polymorphic	KX261868
		R‐AGATCCAGGAGCGAAGAACA						
Mmu14	12929751 492 1089	F‐ACCGCTTGCTTCTGTTGAGT	(AGC)_6_	186	58	*Callorhinchus milii*	Polymorphic	KX261869
		R‐TCGCACAGACTGATTGAAGG						
Mmu15	14824632	F‐CACCTGATTGAGCAGGAGGT	(CTC)_5_	173	58	*Squalus acanthias*	Monomorphic	KX261870
		R‐TATGGAGGTTGGGATTGCAG						

Annealing temperature in °C (*T*_A_).

**Table 3:** Characteristics of two polymorphic microsatellite multiplex assays for *Mustelus mustelus* based on two sampling ocean basins in South Africa, Southeast Atlantic Ocean (SEAO) and Southwest Indian Ocean (SWIO)

Locus	Microsatellite repeat motif	[P]	Dye	Ocean basin	*N*	Size range (bp)	*N*_A_	*A*_R_	*H*_O_	*H*_E_	PIC	*F*_IS_	*P*_HW_	*Fr*_NULL_
Mmu2	(AC)_6_	0.2	FAM	SEAO	25	150–180	5	4.8	0.96	0.68	0.63	−0.391	0.000	0.000
				SWIO	23		4	3.5	0.65	0.48	0.41	−0.352	0.495	0.000
Mmu3	(TC)_7_	0.2	NED	SEAO	25	230–250	4	3.6	0.48	0.54	0.48	0.127	0.643	0.005
				SWIO	22		6	5.2	0.41	0.65	0.59	0.393	0.054	0.121
Mmu4	(TG)_7_	0.2	VIC	SEAO	25	158–180	8	6.3	0.88	0.67	0.61	−0.297	0.000	0.025
				SWIO	23		4	3.5	0.61	0.48	0.41	−0.260	0.368	0.000
Mmu8	(CAG)_5_(TGT)_5_	0.3	VIC	SEAO	25	417–440	8	7.1	0.72	0.78	0.75	0.096	0.710	0.016
				SWIO	21		8	6.8	0.62	0.79	0.76	0.240	0.416	0.094
Mmu11	(CAA)_5_	0.3	PET	SEAO	24	203–209	3	3.0	0.75	0.62	0.55	−0.181	0.033	0.000
				SWIO	16		4	3.9	0.69	0.69	0.63	0.035	0.352	0.000
Mmu13	(GCA)_5_	0.2	NED	SEAO	25	85–109	4	3.6	0.96	0.63	0.57	−0.506	0.001	0.000
				SWIO	23		5	4.5	0.78	0.57	0.53	−0.349	0.485	0.000
MPS1 (mean)	**–**	**–**	**–**			**–**	**6.5**	**5.6**	**0.714**	**0.651**	**0.588**	**−0.097**	**–**	**0.027**
Mmu1	(AT)_7_	0.2	VIC	SEAO	24	200–216	5	4.9	1.00	0.69	0.64	−0.428	0.072	0.000
				SWIO	24		6	5.2	0.92	0.66	0.62	−0.369	0.443	0.000
Mmu5	(CTC)_6_	0.3	FAM	SEAO	24	268–274	2	2.0	0.12	0.50	0.37	0.759	0.000	0.250
				SWIO	22		4	3.2	0.23	0.53	0.43	0.590	0.067	0.193
Mmu6	(CGC)_6_	0.3	PET	SEAO	24	204–214	5	4.2	0.38	0.36	0.33	−0.035	0.000	0.000
				SWIO	21		5	4.5	0.62	0.59	0.55	−0.022	0.040	0.020
Mmu7	(GCT)_5_	0.2	NED	SEAO	23	203–217	3	2.2	0.09	0.08	0.08	−0.011	0.997	0.000
				SWIO	24		6	5.2	0.50	0.52	0.49	0.061	0.143	0.000
Mmu14	(AGC)_6_	0.2	FAM	SEAO	24	160–180	5	4.6	0.38	0.70	0.64	0.480	0.003	0.189
				SWIO	24		5	4.6	0.50	0.70	0.65	0.310	0.008	0.114
MPS2 (mean)	**–**	**–**	**–**			**–**	**5.8**	**4.9**	**0.471**	**0.554**	**0.496**	**0.150**	**–**	**0.078**
Overall (mean)	**–**	**–**	**–**			**–**	**6.2**	**5.3**	**0.604**	**0.607**	**0.546**	**0.005**	**–**	**0.050**

Primer concentration in the final reaction as μmol/L per primer ([P]); number of individuals (*N*); number of alleles per locus (*N*_A_); allelic richness (*A*_R_); observed heterozygosity (*H*_O_); expected heterozygosity (*H*_E_); polymorphic information content (PIC); probability of conformity to Hardy–Weinberg expectations (*P*_HW_); null allele frequency (*Fr*_NULL_). Mean values for each multiplex assay and overall are indicated in bold.

To evaluate the reliability of using cross‐amplified microsatellites for species identification, we conducted multivariate clustering analysis using the discriminant analysis of principal components (DAPC) implemented in the R package ADEGENET (Jombart, 2008). Unlike the Bayesian clustering methods DAPC does not require specific genetic assumptions for the loci used (*e*.*g*., Hardy–Weinberg and linkage equilibria). We only focused on the four coastal sharks that are commonly misidentified in South African fisheries, the common smooth‐hound, spotted gully shark, tope shark, and the whitespotted smooth‐hound. We performed the DAPC analysis on clusters defined by species and assessed the assignment of each individual to distinct genetic clusters using the membership coefficient, *i*.*e*., the percentage of the genotype's ancestry attributed to each genetic cluster. For successful species identification, membership coefficient values had to be ≥95%.

### 2.4 Microsatellite characterization

For the four study species, we tested all loci for scoring errors and allelic dropout using MICRO‐CHECKER v. 2.2.3 (van Oosterhout, Hutchinson, Wills, & Shipley, 2004). The Microsatellite Excel Toolkit (MSATTOOLS v. 1.0, Park, 2001) was used to identify samples sharing identical multilocus genotypes. Duplicate genotypes with ≥95% matching alleles were excluded from further analyses. Using FREENA (Chapuis & Estoup, 2007), we estimated the frequency of null alleles following the expectation maximization (EM) method described by Dempster, Laird, and Rubin (1977). We calculated deviations from Hardy–Weinberg equilibrium (HWE) for each locus using the exact probability test based on 10,000 iterations (10,000 dememorization, 500 batches) in GENEPOP v. 4.0 (Rousset, 2008). We assessed linkage disequilibrium among loci using an exact test, also implemented in GENEPOP. False discovery rate (FDR; Benjamini & Yekutieli, 2001) control was used to adjust *p*‐values for multiple comparisons (i.e., tests for departure from HWE and linkage disequilibrium) to minimize type I errors (see Narum, 2006). To test for potential signatures of selection for each locus, we used LOSITAN v. 1.44 (Antao, Lopes, Lopes, Beja‐Pereira, & Luikart, 2008) with 200,000 simulations following the *F*_ST_ outlier method of Beaumont and Nichols (1996).

### 2.5 Within‐species population genetic analysis

Across sampling sites and species, we calculated the mean number of alleles per locus (*N*_A_), allelic richness standardized for small sample size (*A*_R_), observed heterozygosity (*H*_O_), and heterozygosity expected under conditions of Hardy–Weinberg equilibrium (*H*_E_) using the DIVERSITY (Keenan, McGinnity, Cross, Crozier, & Prodöhl, 2013) package for R (R Development Core Team 2015). We used MSATTOOLS to calculate the polymorphic information content (PIC) according to the equation described in Botstein, White, Skolnick, and Davis (1980). The inbreeding coefficient (*F*_IS_) was calculated in ARLEQUIN v. 3.5 (Excoffier & Lischer, 2010) and tested for deviations from zero using a permutation test (1,000 permutations) with significance values adjusted using the FDR correction for multiple tests. We then used POWSIM v. 4.1 (Ryman & Palm, 2006) to assess the statistical power of the loci for *F*_ST_ tests (i.e., rejection of the null hypothesis *H*_0_ of genetic homogeneity among two subpopulations when it is false) and the α level (i.e., rejection of *H*_0_ when it is true) using a sampling scheme of two subpopulations with 20 individuals each. The analyses were conducted using 10,000 dememorizations, 100 batches, and 1,000 iterations per batch with the allele frequencies observed for the complete dataset of 11 microsatellite loci and our reported sample sizes for each species.

Pairwise *F*_ST_ (Weir & Cockerham, 1984) and Jost's *D*_est_ (Jost, 2008) were calculated using the DIVERSITY package, and the analysis of molecular variance (AMOVA) was calculated using ARLEQUIN. To account for our sampling strategy, the measures of genetic differentiation comparisons were considered significant if the lower CI was >0, and *p*‐values were <.05 following FDR correction. To visualize population distinctness, we used ADEGENET to perform discriminant analysis of principal components (DAPC) on clusters defined by ocean basin. The number of clusters was assessed using the *find*.*clusters* function, which runs successive *K*‐means clustering with increasing number of clusters (*k*). For selecting the optimal *k*, we applied the Bayesian information criterion (BIC) for assessing the best supported model, and therefore the number and nature of clusters, as recommended by Jombart, Devillard, and Balloux (2010). DAPC scatter plots were only drawn for *k *>* *2. We also used a Bayesian clustering model‐based method implemented in STRUCTURE 2.3 (Pritchard, Stephens, & Donnelly, 2000) to detect the most probable number of genetic clusters (*K*) present in each species. We applied an admixture model with correlated allele frequencies for 10 replicates across *K *=* *1 to *K *=* *10 with each run consisting of 1,000,000 Markov chain Monte Carlo (MCMC) iterations and an initial burn‐in phase of 100,000 iterations assuming no prior population information. Given that only two groups of samples were compared for each species, the *ad hoc* statistic ∆*K* described in Evanno, Regnaut, and Goudet (2005) and commonly used to identify the likely number of genetic clusters was not considered appropriate for our study. This ∆*K* statistic never assigns *K *=* *1 (Evanno et al., 2005). Here, the posterior probability of the data (*X*) for a given *K*, Pr(*X*|*K*), calculated by STRUCTURE was used to compute the mean likelihood *L*(*K*) over 10 runs for each *K* to identify the likely *K* for which *L*(*K*) was highest (Pritchard et al., 2000) as implemented in STRUCTURE HARVESTER 0.6.94 (Earl & vonHoldt, 2012). CLUMPAK (Kopelman, Mayzel, Jakobsson, Rosenberg, & Mayrose, 2015) was used for the graphical representations of the STRUCTURE results. Given that we were uncertain about sampling locations of several individual *Mustelus palumbes*, we also used the program GENECLASS2 v2.0 (Piry et al., 2004), to examine genetic structure based on assignment tests for this species. Assignment probabilities of individuals were calculated using a Bayesian procedure (Rannala & Mountain, 1997) and Monte Carlo resampling using 100,000 simulated individuals and a threshold of 0.01.

Finally, we used the coalescence‐based method in the program MIGRATE‐N 3.6.11 (Beerli, 2006; Beerli & Palczewski, 2010) implemented on the CIPRES Portal v3.3 at the San Diego Supercomputer Center (Miller, Pfeiffer, & Schwartz, 2010) to compare alternative migration pattern across oceans. We evaluated four migration models: (1) a full model with two population sizes and two migration rates (from SEAO to SWIO and from SWIO to SEAO); (2) a model with two population sizes and one migration rate to SEAO; (3) a model with two population sizes and one migration rate to SWIO; (4) a model where SEAO and SWIO are part of the same panmictic population. The mutation‐scaled effective population size *Θ *=* *4*N*_e_μ, where *N*_e_ is the effective population size and μ is the mutation rate per generation per locus, and mutation‐scaled migration rates *M *= *m*/μ, where *m* is the immigration rate per generation, among populations were also calculated in MIGRATE‐N. A Brownian process was used to model microsatellite mutations. The Metropolis–Hastings algorithm was used to sample from the prior distributions and generate posterior distributions. Each model was run using random genealogy and values of the parameters *Θ* and *M* produced by *F*_ST_ calculation as a start condition. Bayesian search strategy was conducted using the following parameters: an MCMC search of 5 × 10^5^ burn‐in steps followed by 5 × 10^6^ steps with parameters recorded every 20 steps. The prior distribution for the parameters was uniform with *Θ* and migration boundaries defined after explorative runs. A static heating scheme with four different temperatures (1.0, 1.5, 3.0, and 1 × 10^6^) was employed, where acceptance–rejection swaps were proposed at every step. The model comparison was made using log‐equivalent Bayes factors (LBF) that need the accurate calculation of marginal likelihoods. These likelihoods were calculated using thermodynamic integration in MIGRATE‐N. Models were ordered by LBF, and the model probability (*P*_Mi_) was calculated in R. Additionally, we converted estimates of gene flow (*M*) to the number of effective migrants (*N*_*e*_ *m*) from population *i* to population *j* using the formula:

$$N_{e}^{\left(j\right)}m_{i\to j}=\frac{\Theta_{j}M_{i\to j}}{4}$$

## 3 Results

### 3.1 Microsatellite multiplex assays, cross‐species amplification, and species identification

**Figure A1:**
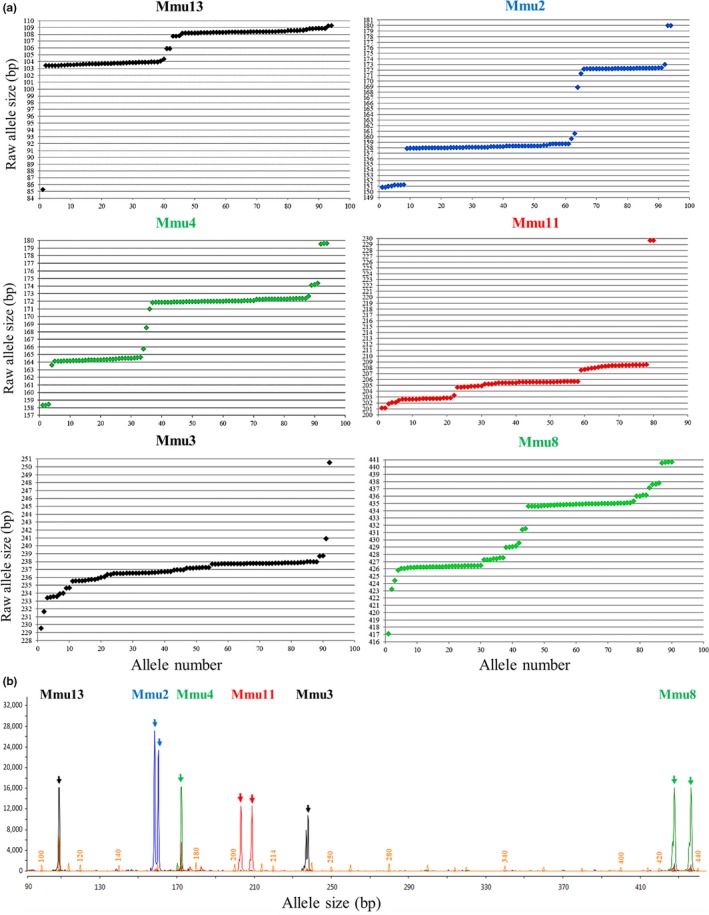
Binning and profiles for multiplex 1. (a) Allelograms based on 48 individuals from the South African South‐East Atlantic and South‐West Indian Ocean sampled populations, respectively. Here, the allele number corresponds to the ranking number of the allele in the list of allele raw sizes, ranked in increasing order. (b) Example of an individual electropherograms where arrows point to alleles at each locus, and small peaks with numbers (base pairs) correspond to fragments of the internal size standard LIZ600

**Figure A2:**
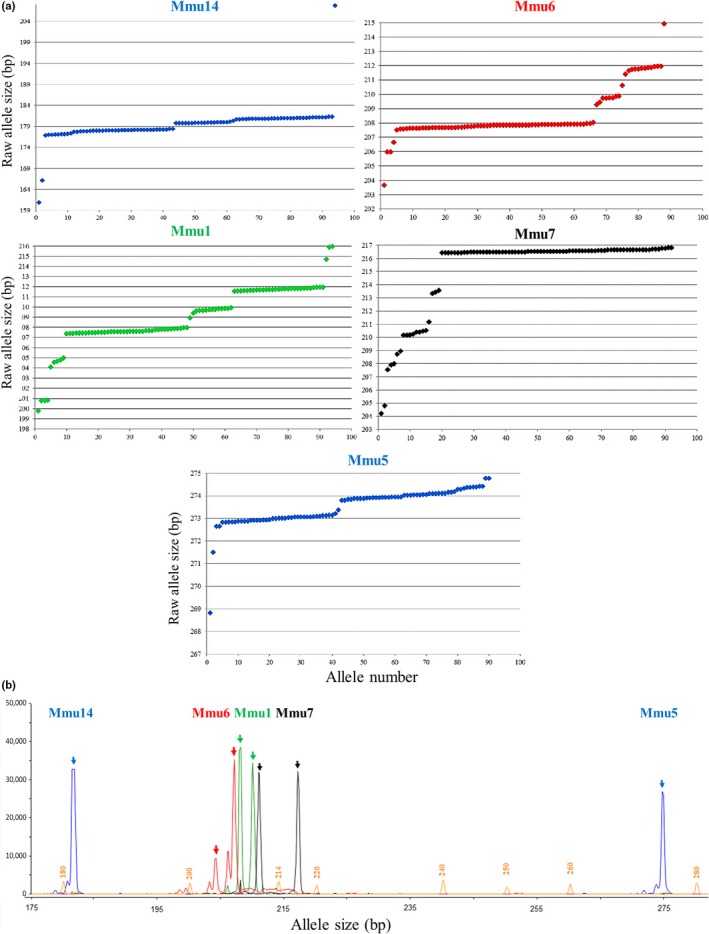
Binning and profiles for multiplex 2. (a) Allelograms based on 48 individuals from the South African South‐East Atlantic and South‐West Indian Ocean sampled populations, respectively. Here, the allele number corresponds to the ranking number of the allele in the list of allele raw sizes, ranked in increasing order. (b) Example of an individual electropherograms where arrows point to alleles at each locus, and small peaks with numbers (base pairs) correspond to fragments of the internal size standard LIZ600

The two sequencing runs of the Nextera™ library for *Mustelus mustelus* generated 35 GB of raw reads. After trimming the raw sequences that included removal of adapters, N‐containing reads, and low‐quality reads, we retained a total of 17 GB clean reads. After the *de novo* assembly of the Illumina paired‐end reads, we recovered a total of 27,512,666 contigs. We identified a total of 82,879 contigs that were longer than 250 bp, of which 2,572 (3.1%) contained microsatellites. Dinucleotide repeats were the most frequent (1,629 or 86.1%), followed by trinucleotide repeats (232 or 12.3%), and tetranucleotide repeats (31 or 1.6%). We selected 15 microsatellite containing contigs for primer design with an expected PCR product size ranging between 112 and 431 bp. Of the 15 loci tested, all were successfully amplified while only 11 were polymorphic based on initial screening via polyacrylamide gels (Table 2). These loci were fluorescently labeled to construct a 5‐plex and 6‐plex assay that were both validated over 48 individuals from two populations of the common smooth‐hound (Figures A1 and A2, Appendix).

The genetic diversity summary statistics for both multiplex assays are presented in Table 2. All markers were polymorphic and produced a total of 74 alleles (mean 6.2). There was no evidence of stutter products or significant allelic dropout based on the MICRO‐CHECKER results, but null alleles were detected at two loci (Mmu5 and Mmu14) with high frequencies estimated in FREENA relative to the rest of the loci (Table 3). After correcting for multiple tests, all loci were in agreement with HWE except for Mmu5 and Mmu14 possibly due to null alleles. Linkage disequilibrium was not found between any of the loci pairs tested. The *F*_ST_‐outlier test showed that locus Mmu7 did not conform to selective neutrality and was under putative directional selection. The PIC ranged from 0.08 to 0.76, and the *H*_O_ and *H*_E_ ranged from 0.09 to 1 and 0.08 to 0.79, respectively. The *F*_IS_ value ranged from −0.506 to 0.759. Subsequent estimates of population genetic structure were therefore computed using a subset of eight microsatellites, excluding loci not conforming to Hardy–Weinberg equilibrium, neutrality, and/or exhibiting high null allele frequencies (Mmu5, Mmu7, and Mmu14). To assess the cross‐species utility of the two multiplexes, we tested these assays on six other triakid species, and cross‐species amplification rate of success ranged from 72% to 100% (Table 4).

**Table 4:** Cross‐species transferability results of 11 microsatellites tested among six triakid species

Locus/species	*Galeorhinus galeus* (*N *=* *8)	*Mustelus asterias* (*N *=* *3)	*M. mosis* (*N *=* *2)	*M. palumbes* (*N *=* *8)	*M. punctulatus* (*N *=* *3)	*Triakis megalopterus* (*N *=* *8)
Mmu1	+ (3)	+ (1)	+ (4)	+ (3)	+ (4)	+ (2)
Mmu2	+ (3)	+ (2)	+ (1)	+ (2)	+ (1)	+ (2)
Mmu3	+ (2)	+ (3)	+ (3)	+ (2)	+ (3)	+ (2)
Mmu4	+ (2)	+ (4)	+ (2)	+ (3)	+ (4)	+ (2)
Mmu5	+ (2)	+ (2)	+ (1)	+ (2)	+ (2)	+ (2)
Mmu6	+ (2)	+ (1)	+ (1)	+ (2)	–	+ (2)
Mmu7	+ (2)	+ (1)	+ (2)	+ (3)	–	+ (2)
Mmu8	+ (4)	+ (2)	+ (1)	+ (5)	+ (2)	+ (2)
Mmu11	+ (2)	+ (1)	+ (1)	+ (2)	+ (3)	+ (2)
Mmu13	+ (3)	+ (2)	+ (2)	+ (3)	+ (6)	+ (3)
Mmu14	+ (2)	+ (2)	+ (3)	+ (2)	–	+ (2)

–, no visible band or faint bands with insufficient band intensity for scoring alleles were observed; +, solid bands with sufficient intensity for scoring alleles were detected, and in brackets the number of alleles per locus are shown.

Additionally, to validate the potential of these markers for within‐species population genetic analysis, we inferred genetic variation in samples collected from two different ocean basins for each respective species (Table 1). In each species, all 11 microsatellites were variable with up to a mean *N*_A_ of 8, *A*_R_ up to 7.5, *H*_E_ and PIC as high as 0.842 (Tables A1, A3, and A3, Appendix). After correcting for multiple tests, all loci in each species conformed to HWE and no evidence for LD between any of the loci pairs were found. MICRO‐CHECKER indicated the presence of null alleles at locus Mmu11 for the tope shark and locus Mmu4 for the spotted gully shark. Using the *F*_ST_‐outlier test, we only found evidence for two loci (Mmu 2 and Mmu11) putatively subjected to selection in the whitespotted smooth‐hound possibly due to issues surrounding small sample sizes. Assessment of the power of the multilocus dataset to detect population structure indicated that all loci used could accurately detect differentiation as low as *F*_ST_ **=* *0.003, for a population sample of *n *=* *20, indicating that the dataset was suitable for population structure inference.

**Figure 3:**
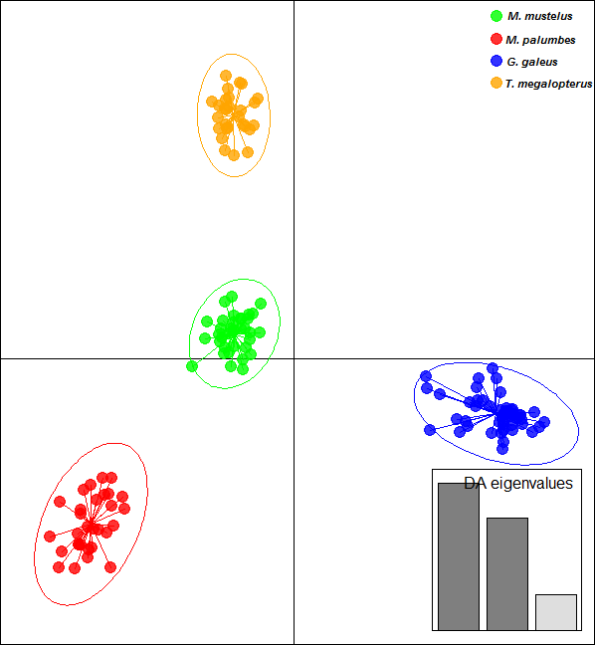
Scatterplots of DAPC analysis for a global picture of the clusters composition between species. The graph represents the individuals as dots and the groups as inertia ellipses. Eigenvalues of the analysis are displayed in inset

The novel microsatellite loci demonstrated potential application in the identification of the study species. The results from the multivariate clustering analysis (DAPC) clearly depict four genetic clusters representative of each species with limited overlap (Figure 3). Here, individuals assigned to one of the four genetic clusters with a membership coefficient of >95%.

### 3.2 Population genetic structure and gene flow

#### 3.2.1 Common smooth‐hound *Mustelus mustelus*

**Figure 4:**
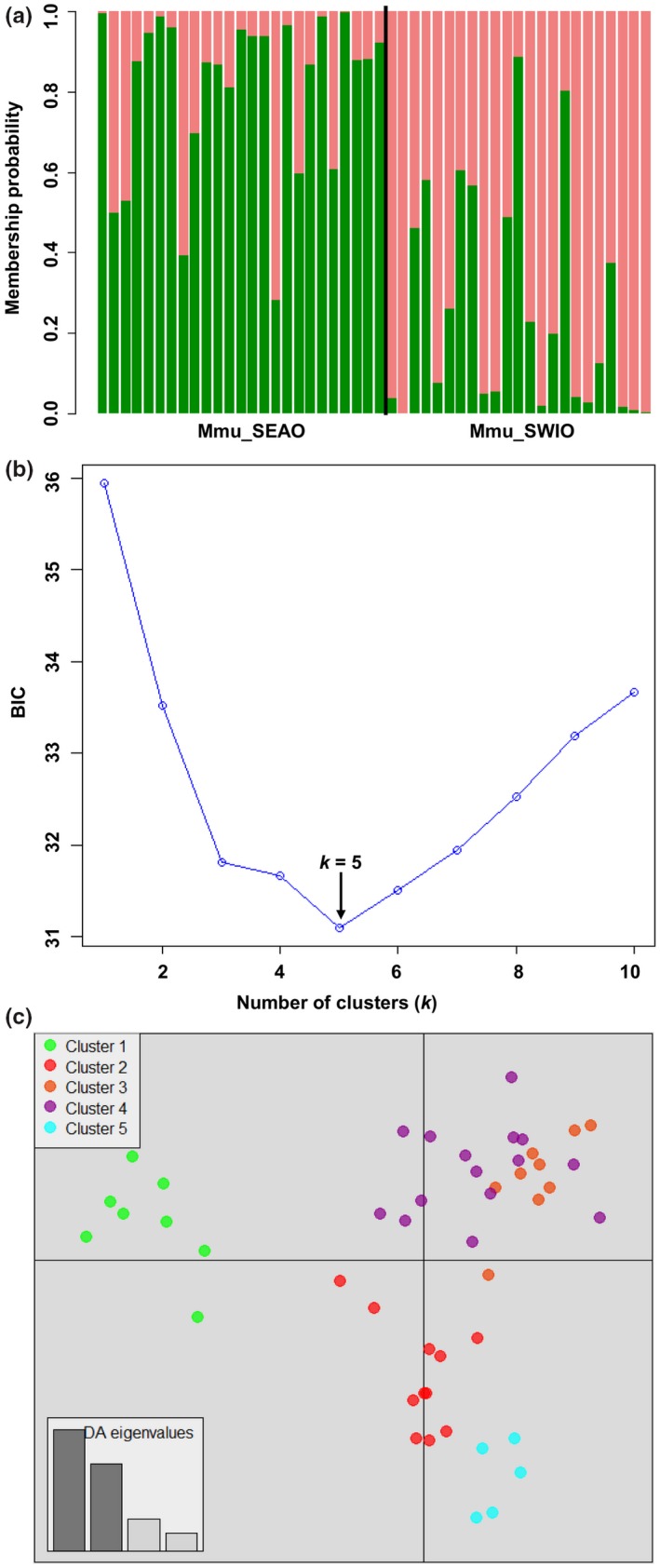
STRUCTURE‐like plot, inference of the number of clusters, and scatterplots of DAPC analysis on the dataset of *Mustelus mustelus*. Mmu_SEAO and Mmu_SWIO represent the South African Southeast Atlantic and Southwest Indian Ocean sampled populations, respectively. (a) Cluster assignments by population (sampling location a priori), each individual is represented by a vertical colored line. (b) Inference of the number of clusters excluding sampling location as a priori. A *k* value of 5 (the lowest BIC value) represents the best summary of the data. (c) The graph represents the individuals as dots. Each color represents a genetic cluster (*k*)

**Figure A3:**
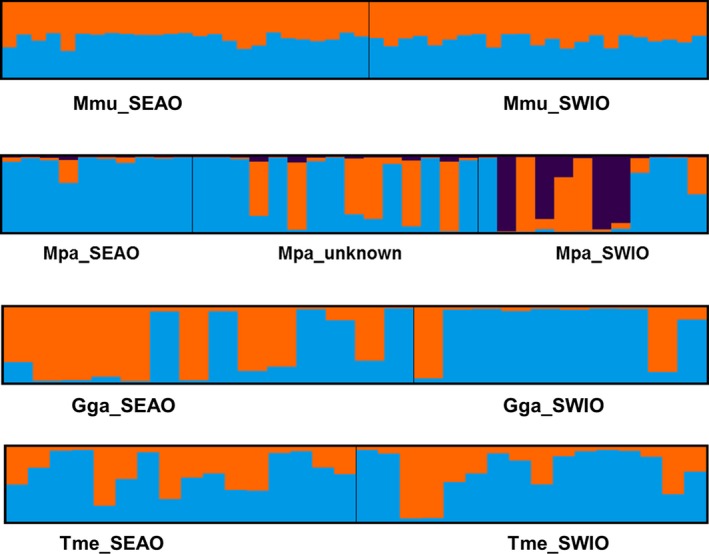
STRUCTURE results showing the most likely number of genetic clusters present in each of the four study species. SEAO and SWIO represents the South African South‐East Atlantic and South‐West Indian Ocean samples, respectively. Bar plots showing individual genotype membership to K clusters (each cluster is represented by a different colour, and each vertical bar represents an individual)

**Figure A4:**
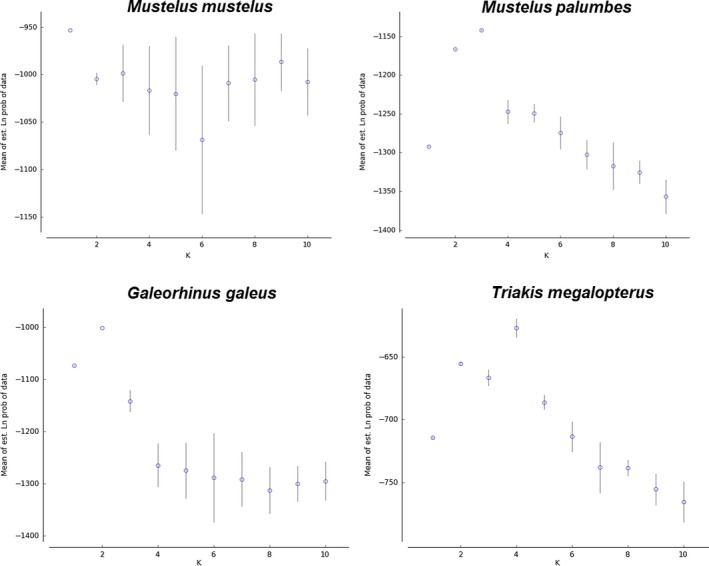
Likelihood probability profile estimated from STRUCTURE at K1‐10 showing the mean and variance at each K for each study species.

The pairwise population differentiation indices (*F*_ST_ **=* *0.029, *D*_est_ **=* *0.021) and AMOVA (*F*_ST_ **=* *0.029, Table A4) indicated the presence of shallow population genetic structure between SEAO and SWIO (i.e., lower 95% confidence intervals >0, and *p*‐values <.05 after FDR corrections). The DAPC analysis including location prior revealed two clear genetic clusters corresponding to ocean basins, whereas excluding location prior using the *find*.*clusters* function, the DAPC analysis identified the presence of five genetic clusters (*k *=* *5) in the dataset based on the BIC score (Figure 4). The postprocessing of the STRUCTURE results using *L*(*K*) revealed one admixed cluster (*K *=* *1) as the most likely number of groups present in the dataset (Figures A3a and A4a, Appendix). Coalescent analyses for migration model comparison highly supported (*P*_Mi_ **=* *1.0) Model 2 (*i*.*e*., migration from SWIO to SEAO) and showed that Θ was highest in the SWIO (Θ = 5.870) and lowest in the SEAO (Θ = 0.790) (Tables A5 and A6).

#### 3.2.2 Whitespotted smooth‐hound *Mustelus palumbes*

**Figure 5:**
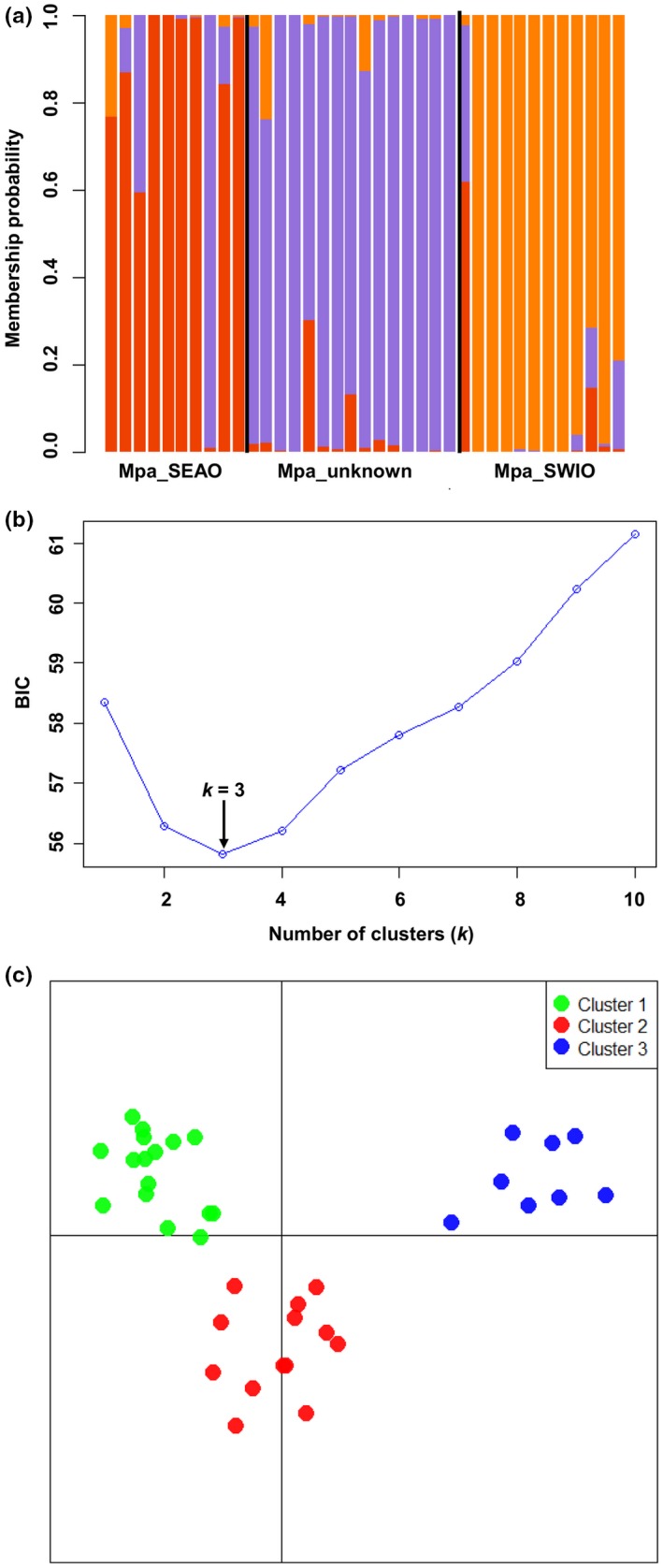
STRUCTURE‐like plot, inference of the number of clusters, and scatterplots of DAPC analysis on the dataset of *Mustelus palumbes*. Mpa_SEAO and Mpa_SWIO represent the South African Southeast Atlantic and Southwest Indian Ocean sampled populations, respectively. (a) Cluster assignments by population (sampling location a priori), each individual is represented by a vertical colored line. (b) Inference of the number of clusters excluding sampling location as a priori. A *k* value of 3 (the lowest BIC value) represents the best summary of the data. (c) The graph represents the individuals as dots. Each color represents a genetic cluster (*k*)

Pairwise differentiation test using *F*_ST_ indicated significant population differentiation estimates, which were congruent with the results obtained with Jost's *D*_est_ between all putative populations. Pairwise comparison of the unknown samples (in terms of sampling region) with the samples collected from the SEAO revealed low differentiation (*F*_ST_ **=* *0.021, *D*_est_ **=* *0.017, lower 95% CI > 0), higher levels when compared with the SWIO samples (*F*_ST_ **=* *0.086, *D*_est_ **=* *0.104, lower 95% CI > 0). Notably, population differentiation estimates were significantly large for Atlantic versus Indian Ocean comparisons (*F*_ST_ **=* *0.091, *D*_est_ **=* *0.155, lower 95% CI > 0). Global AMOVA results indicated within individual variation explains a greater amount of the total genetic variation, with less variation among populations (*F*_ST_ **=* *0.069, *p *<* *.01) (Table A4). The DAPC analysis including and excluding the location prior revealed three genetic clusters (*k *=* *3) in the dataset based on the BIC score (Figure 5). Individual assignment test based on a Bayesian approach for mapping the origin of the unknown putative population assigned 60% of the individuals to the SEAO and the remainder to the SWIO, indicative of the possible existence of substructure in *M*. *palumbes*. Bayesian clustering analysis in STRUCTURE also supported the assignment of the unknown population to the SEAO and interoceanic population subdivision (Figures A3b and A4b, Appendix). The most likely number of groups present in the data was *K *=* *3. All results were considered, we assumed the unknown putative population to have been sampled from the SEAO, and therefore, for the gene flow analysis, we grouped the unknown samples with the samples from the SEAO. The most probable migrate‐N coalescent model of population structure was the unidirectional model assuming asymmetric migration from SWIO to SEAO (*P*_Mi_ **=* *1.0). Estimates of Θ was highest in the SWIO (Θ = 19.660) and lowest in the SEAO (Θ = 0.540) (Tables A5 and A6).

#### 3.2.3 Tope shark *Galeorhinus galeus*

**Figure 6:**
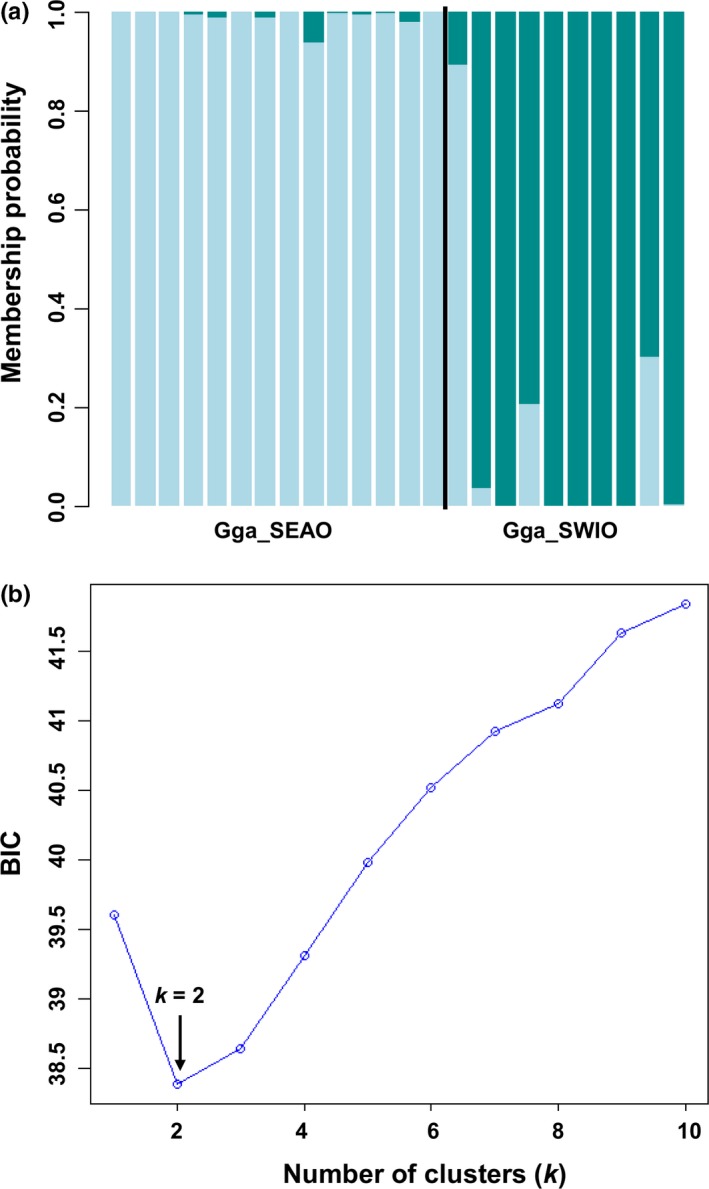
STRUCTURE‐like plot, inference of the number of clusters, and scatterplots of DAPC analysis on the dataset of *Galeorhinus galeus*. Gga_SEAO and Gga_SWIO represent the South African Southeast Atlantic and Southwest Indian Ocean sampled populations, respectively. (a) Cluster assignments by population (sampling location a priori), each individual is represented by a vertical colored line. (b) Inference of the number of clusters excluding sampling location as a priori. A *k* value of 2 (the lowest BIC value) represents the best summary of the data. Each color represents a genetic cluster (*k*)

Population differentiation between the SEAO and SWIO was significantly greater than zero (*F*_ST_ **=* *0.034, lower 95% CI > 0), while similar to *M. mustelus*, Jost's *D*_est_ indicated less pronounced levels of differentiation (*D*_est_ **=* *0.076, lower 95% CI > 0). The AMOVA results showed that there was no differentiation among populations (*F*_ST_ **=* *0.033, *p *=* *.135), but a significant amount of variance was attributed to among individuals within populations (*F*_IS_ **=* *0.093, *p *=* *.000) and within individuals (*F*_IT_ **=* *0.123, *p *=* *.000) (Table A4). The DAPC analysis including and excluding the location prior revealed two genetic clusters (*k *=* *2) in the dataset based on the BIC score (Figure 6). Evaluation of the *K* values produced by STRUCTURE using the maximum value of *L*(*K*) identified *K *=* *2 as the most likely number of groups present in the data (Figures A3c and A4c, Appendix). Coalescent analyses for migration model comparison highly supported (*P*_Mi_ **=* *1.0) Model 2 (i.e., migration from SWIO to SEAO) and showed that Θ was highest in the SWIO (Θ = 98.100) and lowest in the SEAO (Θ = 0.100) (Tables A5 and A6).

#### 3.2.4 Spotted gully shark *Triakis megalopterus*

**Figure 7:**
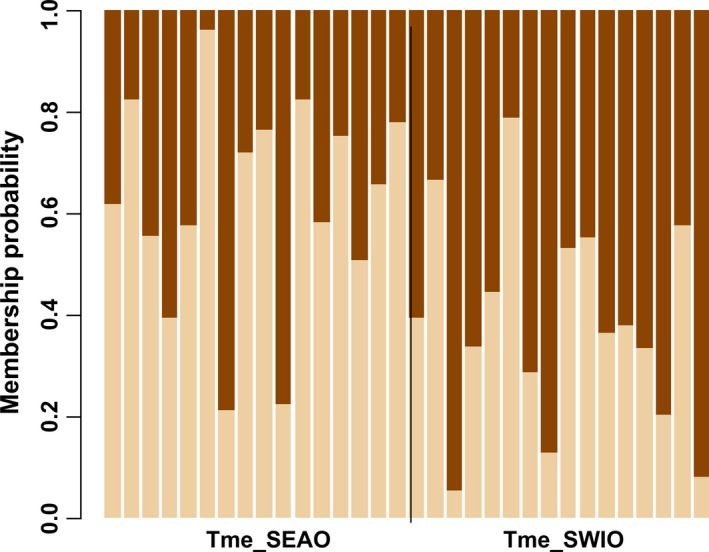
STRUCTURE‐like plot of DAPC analysis on the dataset of *Triakis megalopterus*. Tme_SEAO and Tme_SWIO represent the South African Southeast Atlantic and Southwest Indian Ocean sampled populations, respectively. Each individual is represented by a vertical colored line, and each color represents a genetic cluster (*k*)

Based on the population differentiation estimates, there was no evidence for population subdivision between the SEAO and SWIO samples (*F*_ST_ = −0.012, *D*_est_ **= −0.002, lower 95% CI < 0). The AMOVA results also showed no differentiation among populations (*F*_ST_ = −0.012, *p *=* *1.000), with most of the variation explained among individuals within populations (*F*_IS_ **=* *0.134, *p *=* *.000) and within individuals (*F*_IT_ **=* *0.123, *p *=* *.000) (Table A4). The DAPC analysis showed clustering with fairly flat distributions of membership probabilities of individuals across clusters indicative of one genetic cluster in the data (Figure 7). Bayesian clustering analysis in STRUCTURE identified four admixed genetic clusters (*K *=* *4) as the most likely number of groups present in the data (Figures A3d and A4d, Appendix). Coalescent analyses for migration model comparison highly supported (*P*_Mi_ **=* *1.0) Model 2 (i.e., migration from SWIO to SEAO) and showed that Θ was highest in the SWIO (Θ = 6.820) and lowest in the SEAO (Θ = 1.380) (Tables A5 and A6).

## 4 Discussion

Recent advances in next‐generation sequencing technologies have considerably accelerated the mining of species‐specific microsatellite loci in shark species generally devoid of molecular markers (Blower et al., 2015; Chabot & Nigenda, 2011; Pirog et al., 2015). In this study, the use of Illumina HiSeq^™^ 2000 for reduced genome sequencing was successful regarding speed, accuracy, and cost in generating microsatellites. It provided an efficient way to develop microsatellite markers, even though some factors such as library preparation, read length, and precision of the assembly can be improved in future studies. The relative richness of different types of microsatellite repeats is typical, and in sharks, dinucleotide repeats are generally overrepresented. Similar to the studies of the Australian gummy shark *Mustelus antarticus* (Boomer & Stow, 2010), the tope shark (Chabot & Nigenda, 2011), and the brown smooth‐hound shark *M*. *henlei* (Chabot, 2012), we found that dinucleotide microsatellite repeats were the most frequent repeat type present in the common smooth‐hound shark genome. Furthermore, we successfully constructed and optimized two polymorphic multiplex assays for the common smooth‐hound shark. The validation of our multiplex assays in the common smooth‐hound revealed similar genetic diversity indices as found in a previous study of the same species using cross‐amplified loci (Maduna et al., 2016). Given that in sharks, microsatellite flanking sequences are conserved owing to low mutation rates (Martin, Pardini, Noble, & Jones, 2002) we tested for the cross‐species amplification of orthologous microsatellite loci in other Triakidae species. We observed a high cross‐species amplification rate of success (>70%) across all microsatellite loci. Such findings were similar to those previously reported on sharks (Blower et al., 2015; Chabot & Nigenda, 2011; Giresi, Renshaw, Portnoy, & Gold, 2012).

There is often a negative correlation between the evolutionary distance of the focal and target species, and the transferability of loci (amplification success and polymorphism) in sharks (Maduna et al., 2014). A similar trend has also been found in several other vertebrate taxa including birds, amphibians, and fish (Carreras‐Carbonell, Macpherson, & Pascual, 2007; Hendrix, Susanne Hauswaldt, Veith, & Steinfartz, 2010; Primmer, Painter, Koskinen, Palo, & Merilä, 2005). All the species that were included in this study were closely related and accordingly the high performance of cross‐species amplification was expected, albeit the blackspotted smooth‐hound had the lowest transferability rate possibly due to the presence of null alleles. These loci, nevertheless, could prove useful in elucidating patterns of population genetic structure and gene flow within other Triakidae species. Besides the comparison of population genetic parameters among multiple closely related species, cross‐species microsatellites can also be applied for species identification based on species‐specific allele sizes at multiple loci, a technique that has rarely been used for forensic studies of sharks (Giresi et al., 2015; Maduna et al., 2014; Marino et al., 2014). Indeed, our multiplex assays proved useful in discriminating between the study species, particularly for those that are morphologically very similar.

Our assessment of the distribution of genetic diversity of the four codistributed coastal sharks (the common smooth‐hound, spotted gully shark, tope shark, and the whitespotted smooth‐hound) based on the newly developed multiplex assays indicated that the microsatellite loci are informative for species identification as well as for population genetic analysis. Our preliminary population genetics estimates hinted at the combined effects of oceanographical barriers and life‐history differences (e.g., mobility and sex‐specific dispersal strategies) to be the major factors influencing the patterns of regional population structure in these sharks. We rejected the null hypothesis of panmixia in all the study species except for *T*. *megalopterus*. In line with previous studies by Bitalo et al. (2015) and Maduna et al. (2016), we detected interoceanic genetic structure in the common smooth‐hound across the Atlantic/Indian Ocean boundary. Our findings also suggest the presence of fine‐scale genetic structure in the whitespotted smooth‐hound, indicating that the unknown sampling population was collected along a gradient of restricted gene flow. Based on the Bayesian (STRUCTURE and GENECLASS) and multivariate (DAPC) analyses, it is evident that the majority of the unknown samples came from the Atlantic Ocean. In *Mustelus* species, it seems intraspecific populations are typically connected via a series of stepping stone populations (Boomer, 2013; Pereyra et al., 2010). In such systems genetic structure is usually reflected by a combination of effective population size, individual movements and migrations, seascape feature, and habitat preferences, *e*.*g*., the narrownose smooth‐hound *M*. *schmitti* (Pereyra et al., 2010), the Australian gummy shark (Boomer, 2013), the rig *M*. *lenticulatus* (Boomer, 2013), and the brown smooth‐hound shark (Chabot et al., 2015; Sandoval‐Castillo & Beheregaray, 2015). Pereyra et al. (2010) and Boomer (2013) found no evidence of population genetic structure, while Chabot et al. (2015) and Sandoval‐Castillo and Beheregaray (2015) provided compelling evidence for the interplay of oceanography and dispersal differential between sexes in shaping genetic structure. In agreement with Maduna et al. (2016), our study found asymmetric gene flow that predominantly occurs from the Southwest Indian Ocean to Southeast Atlantic Ocean for the common smooth‐hound, and a similar trend was observed for the whitespotted smooth‐hound. Granted, the reproductive and seasonal behavior of the two study smooth‐hounds remain for the most part unknown (sensu Smale & Compagno, 1997; da Silva et al., 2013), particularly for the whitespotted smooth‐hound, but it appears that genetic structure in these species is highly similar (at least in the samples investigated here).

Results from previous research indicated that levels of gene flow across the Atlantic/Indian Ocean boundary for the tope shark were relatively high (Bitalo et al., 2015), yet we found significant interoceanic genetic structure with two genetic clusters characterized by lower levels of admixture (SEAO and SWIO). The Bitalo et al. (2015) study, however, included only one Indian Ocean population (Struis Bay) in close proximity to the proposed boundary and noted significant population differentiation between this SWIO sampling site and a SEAO sampling site, Robben Island. In addition, Bitalo et al. (2015) did note that overall samples collected west of the Atlantic/Indian Ocean boundary exhibited a more significant level of admixture than those collected east of the boundary. We conclude that the genetic structure observed in our study is in agreement with that of the previous study given our sampling locations for the species. Similarly, for smooth‐hounds, long‐term gene flow estimates between ocean basins were asymmetrical and mainly occur from the Southwest Indian Ocean to Southeast Atlantic Ocean. The homogenous population structure observed here for the spotted gully shark was unexpected, given the available tagging data which indicate possible philopatric behavior for the species, although, it freely travels across the Atlantic/Indian Ocean boundary (Dunlop & Mann, 2014; Soekoe, 2016). However, it is well documented that the Atlantic/Indian Ocean boundary (Benguela Barrier) or transition zone is not fixed and extends from Cape Point (westernmost boundary) to Cape Agulhas (easternmost boundary) depending on the species in question (Teske, Von der Heyden, McQuaid, & Barker, 2011). The former may hold true for the spotted gully shark given our sampling site that we used as a representative of the Atlantic Ocean (Cape Point and Betty's Bay).

Coalescent analyses for migration model comparison highly supported the model of the southward flux of migrants (i.e., migration from SWIO to SEAO) and showed that Θ was highest in the SWIO and lowest in the SEAO populations in all study species. Our finding of similar asymmetric migration patterns in these species might suggest that such patterns arose from the action of shared physical boundaries. Also, water temperature changes have been shown to influence movement of these triakid sharks and other closely related species (Chabot & Allen, 2009; Espinoza, Farrugia, & Lowe, 2011; da Silva et al., 2013; Soekoe, 2016; West & Stevens, 2001). From the perspective of thermal physiology, albeit speculative, individuals from subtropical and/or warm‐temperate bioregions can more easily colonize the cool‐temperate bioregions as opposed to the reverse. Nevertheless, it is apparent that the cold Benguela Current and its interplay with the warm Agulhas Current also influence the patterns of gene flow in these coastal sharks as evident in a variety of other regional coastal fish species (Henriques, Potts, Santos, Sauer, & Shaw, 2014; Henriques, Potts, Sauer, & Shaw, 2012, 2015) as well as passively dispersing marine species (Teske, Bader, & Rao Golla, 2015). Although our population and genetic sampling are limited, the Agulhas Current presents a significant barrier to the northward migration in smaller coastal sharks. In summary, the newly developed multiplex assays will provide valuable molecular tools for species identification, assessing the distribution of genetic diversity and determining the directionality of gene flow, factors which are all vital for the conservation and management of these local exploited shark species.

## Conflict of Interest

None declared.

## Appendix 1

### 1.1

**Table A1:** Characteristics of two polymorphic microsatellite multiplex assays for *Mustelus palumbes* based on two sampling ocean basins in South Africa, Southeast Atlantic Ocean (SEAO) and Southwest Indian Ocean (SWIO)

Locus	Microsatellite repeat motif	[P]	Dye	Ocean basin	*N*	Size range (bp)	*N*_A_	*A*_R_	*H*_O_	*H*_E_	PIC	*F*_IS_	*P*_HW_	*Fr*_NULL_
Mmu2	(AC)_6_	0.2	FAM	SEAO	10	139–181	4	3.3	0.40	0.36	0.33	−0.108	1.000	0.000
				Unknown	15		3	2.3	0.14	0.20	0.19	0.307	0.111	0.000
				SWIO	12		9	6.9	0.67	0.85	0.79	0.225	0.002	0.038
Mmu3	(TC)_7_	0.2	NED	SEAO	10	228–244	5	4.5	0.60	0.70	0.62	0.150	0.025	0.067
				Unknown	15		7	6.1	0.80	0.86	0.81	0.072	0.172	0.022
				SWIO	12		5	4.4	0.60	0.76	0.67	0.217	0.238	0.074
Mmu4	(TG)_7_	0.2	VIC	SEAO	10	157–201	2	2.0	0.30	0.27	0.22	−0.125	1.000	0.000
				Unknown	15		6	4.1	0.80	0.65	0.57	−0.235	0.000	0.018
				SWIO	12		9	6.7	0.83	0.85	0.79	0.022	0.001	0.000
Mmu8	(CAG)_5_(TGT)_5_	0.3	VIC	SEAO	10	410–438	9	7.6	0.80	0.87	0.81	0.089	0.368	0.031
				Unknown	15		2	8.0	0.85	0.90	0.85	0.057	0.525	0.000
				SWIO	12		3	7.3	0.50	0.88	0.83	0.443	0.000	0.177
Mmu11	(CAA)_5_	0.3	PET	SEAO	10	203–212	11	1.9	0.00	0.19	0.16	1.000	0.053	0.204
				Unknown	15		1	1.0	0.00	0.00	0.00	N.A.	N.A.	0.001
				SWIO	12		7	4.4	0.10	0.74	0.65	0.871	0.000	0.353
Mmu13	(GCA)_5_	0.2	NED	SEAO	10	85–118	9	3.0	0.43	0.67	0.55	0.379	0.329	0.135
				Unknown	15		5	4.8	0.73	0.67	0.61	−0.096	0.203	0.026
				SWIO	12		7	5.3	0.50	0.71	0.65	0.309	0.014	0.057
MPS1 (mean)	–	–	–			**–**	**5.8**	**4.7**	**0.503**	**0.619**	**0.561**	**0.210**	**–**	**0.067**
Mmu1	(AT)_7_	0.2	VIC	SEAO	10	180–214	2	2.0	0.20	0.53	0.38	0.633	0.075	0.200
				Unknown	15		6	4.8	0.73	0.71	0.65	−0.037	0.133	0.000
				SWIO	12		6	5.4	0.50	0.82	0.75	0.400	0.005	0.168
Mmu5	(CTC)_6_	0.3	FAM	SEAO	10	253–291	3	2.9	0.30	0.62	0.49	0.526	0.061	0.199
				Unknown	15		6	4.5	0.54	0.66	0.60	0.196	0.159	0.012
				SWIO	12		7	5.3	0.58	0.77	0.70	0.252	0.001	0.103
Mmu6	(CGC)_6_	0.3	PET	SEAO	10	203–213	4	4.0	0.50	0.76	0.67	0.357	0.034	0.124
				Unknown	15		6	5.0	0.64	0.73	0.67	0.127	0.611	0.018
				SWIO	12		6	5.7	0.56	0.81	0.74	0.328	0.012	0.129
Mmu7	(GCT)_5_	0.2	NED	SEAO	10	201–219	4	3.5	0.56	0.58	0.48	0.036	1.000	0.000
				Unknown	15		6	4.7	0.64	0.62	0.57	−0.045	0.555	0.000
				SWIO	12		8	6.5	0.92	0.85	0.79	−0.080	0.071	0.000
Mmu14	(AGC)_6_	0.2	FAM	SEAO	10	163–209	4	3.8	0.40	0.67	0.58	0.419	0.013	0.147
				Unknown	15		6	4.7	0.67	0.74	0.67	0.097	0.183	0.028
				SWIO	12		12	9.2	0.83	0.93	0.88	0.109	0.125	0.044
MPS2 (mean)	**–**	**–**	**–**			**–**	**5.7**	**4.8**	**0.571**	**0.720**	**0.641**	**0.221**	**–**	**0.078**
Overall (mean)	**–**	**–**	**–**			**–**	**5.8**	**4.7**	**0.534**	**0.665**	**0.597**	**0.215**	**–**	**0.072**

Primer concentration in the final reaction as μmol/L per primer ([P]); number of individuals (*N*); number of alleles per locus (*N*_A_); allelic richness (*A*_R_); observed heterozygosity (*H*_O_); expected heterozygosity (*H*_E_); polymorphic information content (PIC); probability of conformity to Hardy–Weinberg expectations (*P*_HW_); null allele frequency (*Fr*_NULL_). Mean values for each multiplex assay and overall are indicated in bold.

**Table A2:** Characteristics of two polymorphic microsatellite multiplex assays for *Galeorhinus galeus* based on two sampling ocean basins in South Africa, Southeast Atlantic Ocean (SEAO) and Southwest Indian Ocean (SWIO)

Locus	Microsatellite repeat motif	[P]	Dye	Ocean basin	*N*	Size range (bp)	*N*_A_	*A*_R_	*H*_O_	*H*_E_	PIC	*F*_IS_	*P*_HW_	*Fr*_NULL_
Mmu2	(AC)_6_	0.2	FAM	SEAO	14	126–172	9	6.9	0.57	0.80	0.75	0.295	0.006	0.101
				SWIO	10		9	8.4	0.90	0.91	0.85	0.012	0.164	0.000
Mmu3	(TC)_7_	0.2	NED	SEAO	14	230–248	7	5.8	0.79	0.79	0.72	0.000	0.104	0.000
				SWIO	10		5	5.0	1.00	0.80	0.72	−0.268	0.064	0.000
Mmu4	(TG)_7_	0.2	VIC	SEAO	14	162–176	5	4.6	0.71	0.74	0.67	0.037	0.004	0.000
				SWIO	10		8	7.3	0.90	0.87	0.80	−0.038	0.081	0.000
Mmu8	(CAG)_5_(TGT)_5_	0.3	VIC	SEAO	11	405–436	10	8.5	0.82	0.90	0.84	0.095	0.007	0.000
				SWIO	8		9	9.0	1.00	0.93	0.85	−0.087	0.218	0.000
Mmu11	(CAA)_5_	0.3	PET	SEAO	13	198–236	8	6.4	0.38	0.73	0.68	0.485	0.000	0.179
				SWIO	10		11	9.7	0.60	0.93	0.87	0.365	0.002	0.147
Mmu13	(GCA)_5_	0.2	NED	SEAO	14	89–124	8	6.7	0.93	0.81	0.76	−0.154	0.394	0.000
				SWIO	10		6	5.2	0.70	0.57	0.52	−0.235	1.000	0.000
MPS1 (mean)	**–**	**–**	**–**			**–**	**7.9**	**7.0**	**0.78**	**0.81**	**0.75**	**0.042**		**0.036**
Mmu1	(AT)_7_	0.2	VIC	SEAO	14	192–218	10	7.8	0.79	0.88	0.83	0.106	0.571	0.000
				SWIO	10		9	7.8	0.90	0.84	0.78	−0.073	0.045	0.000
Mmu5	(CTC)_6_	0.3	FAM	SEAO	14	263–287	7	5.2	0.43	0.65	0.60	0.350	0.032	0.113
				SWIO	10		8	7.7	0.80	0.89	0.83	0.111	0.092	0.036
Mmu6	(CGC)_6_	0.3	PET	SEAO	14	183–220	10	8.1	0.71	0.88	0.83	0.193	0.022	0.035
				SWIO	10		8	7.3	0.60	0.87	0.80	0.321	0.058	0.138
Mmu7	(GCT)_5_	0.2	NED	SEAO	14	200–222	9	6.6	0.64	0.73	0.68	0.127	0.199	0.029
				SWIO	10		10	8.9	0.90	0.89	0.83	−0.013	0.088	0.000
Mmu14	(AGC)_6_	0.2	FAM	SEAO	14	165–211	6	4.5	0.57	0.60	0.54	0.046	0.360	0.000
				SWIO	10		7	6.3	0.70	0.77	0.70	0.094	0.234	0.000
MPS2 (mean)	**–**	**–**	**–**			**–**	**8.4**	**7.0**	**0.70**	**0.80**	**0.74**	**0.126**		**0.035**
Overall (mean)	**–**	**–**	**–**			**–**	**8.14**	**7.00**	**0.74**	**0.81**	**0.75**	**0.080**	** **	**0.035**

Primer concentration in the final reaction as μmol/L per primer ([P]); number of individuals (*N*); number of alleles per locus (*N*_A_); allelic richness (*A*_R_); observed heterozygosity (*H*_O_); expected heterozygosity (*H*_E_); polymorphic information content (PIC); probability of conformity to Hardy–Weinberg expectations (*P*_HW_); null allele frequency (*Fr*_NULL_). Mean values for each multiplex assay and overall are indicated in bold.

**Table A3:** Characteristics of two polymorphic microsatellite multiplex assays for *Triakis megalopterus* based on two sampling ocean basins in South Africa, Southeast Atlantic Ocean (SEAO) and Southwest Indian Ocean (SWIO)

Locus	Microsatellite repeat motif	[P]	Dye	Ocean basin	*N*	Size range (bp)	*N*_A_	*A*_R_	*H*_O_	*H*_E_	PIC	*F*_IS_	*P*_HW_	*Fr*_NULL_
Mmu2	(AC)_6_	0.2	FAM	SEAO	16	153–181	8	7.7	0.94	0.71	0.65	−0.331	0.011	0.000
				SWIO	16		6	5.8	0.75	0.63	0.55	−0.192	0.001	0.023
Mmu3	(TC)_7_	0.2	NED	SEAO	16	230–250	4	3.8	0.19	0.18	0.17	−0.034	1.000	0.000
				SWIO	16		4	3.9	0.19	0.24	0.22	0.211	0.065	0.000
Mmu4	(TG)_7_	0.2	VIC	SEAO	16	164–180	4	3.9	0.19	0.29	0.27	0.357	0.075	0.114
				SWIO	16		4	3.9	0.19	0.29	0.27	0.357	0.075	0.114
Mmu8	(CAG)_5_(TGT)_5_	0.3	VIC	SEAO	16	412–426	2	2.0	0.06	0.42	0.32	0.854	0.002	0.257
				SWIO	16		4	3.9	0.13	0.34	0.31	0.636	0.001	0.146
Mmu11	(CAA)_5_	0.3	PET	SEAO	15	148–238	7	7.0	0.40	0.59	0.54	0.328	0.042	0.123
				SWIO	15		4	4.0	0.40	0.44	0.39	0.102	0.077	0.073
Mmu13	(GCA)_5_	0.2	NED	SEAO	16	100–116	3	2.9	0.75	0.51	0.40	−0.506	0.059	0.000
				SWIO	16		4	4.0	0.88	0.63	0.53	−0.414	0.133	0.000
MPS1 (mean)	**–**	**–**	**–**				**4.5**	**4.4**	**0.42**	**0.44**	**0.38**	**0.114**		**0.071**
Mmu1	(AT)_7_	0.2	VIC	SEAO	16	202–216	3	3.0	0.69	0.64	0.54	−0.082	0.619	0.000
				SWIO	16		7	6.8	0.56	0.76	0.70	0.266	0.037	0.094
Mmu5	(CTC)_6_	0.3	FAM	SEAO	16	271–283	4	3.9	0.13	0.59	0.49	0.794	0.000	0.278
				SWIO	16		4	3.9	0.38	0.59	0.49	0.373	0.102	0.111
Mmu6	(CGC)_6_	0.3	PET	SEAO	16	204–218	6	5.9	0.69	0.68	0.62	−0.015	0.224	0.000
				SWIO	16		6	5.9	0.50	0.76	0.70	0.350	0.009	0.120
Mmu7	(GCT)_5_	0.2	NED	SEAO	16	208–226	6	5.7	0.25	0.29	0.28	0.149	0.305	0.000
				SWIO	16		5	4.9	0.31	0.39	0.36	0.198	0.084	0.000
Mmu14	(AGC)_6_	0.2	FAM	SEAO	16	177–214	5	4.8	0.25	0.24	0.22	−0.053	1.000	0.000
				SWIO	16		4	3.9	0.31	0.29	0.27	−0.087	1.000	0.000
MPS2 (mean)	**–**	**–**	**–**				**5**	**4.87**	**0.41**	**0.52**	**0.47**	**0.189**		**0.060**
Overall (mean)	**–**	**–**	**–**				**4.73**	**4.62**	**0.41**	**0.48**	**0.42**	**0.148**	** **	**0.066**

Primer concentration in the final reaction as μmol/L per primer ([P]); number of individuals (*N*); number of alleles per locus (*N*_A_); allelic richness (*A*_R_); observed heterozygosity (*H*_O_); expected heterozygosity (*H*_E_); polymorphic information content (PIC); probability of conformity to Hardy–Weinberg expectations (*P*_HW_); null allele frequency (*Fr*_NULL_). Mean values for each multiplex assay and overall are indicated in bold.

**Table A4:** Analysis of molecular variance (AMOVA) for *Mustelus mustelus*,* Mustelus palumbes*,* Galeorhinus galeus*, and *Triakis megalopterus*; **p *<* *.05, ***p *<* *.01

Species	Source of variation	Variation (%)	*F* statistic	*p* Value
*Mustelus mustelus*	Among populations	2.9	*F*_ST_ * *=* *0.029	.006**
	Within populations	−13.7	*F*_IS_ * *= −0.147	1.000
	Within individuals	110.8	*F*_IT_ * *=* *0.108	1.000
*Mustelus palumbes*	Among populations	6.9	*F*_ST_ * *=* *0.069	.000**
	Within populations	17.5	*F*_IS_ * *=* *0.188	.000**
	Within individuals	75.6	*F*_IT_ * *=* *0.244	.000**
*Galeorhinus galeus*	Among populations	3.4	*F*_ST_ * *=* *0.033	.135
	Within populations	8.9	*F*_IS_ * *=* *0.093	.000**
	Within individuals	87.7	*F*_IT_ * *=* *0.123	.000**
*Triakis megalopterus*	Among populations	−1.2	*F*_ST_ = −0.012	1.000
	Within populations	13.6	*F*_IS_ * *=* *0.134	.000**
	Within individuals	87.6	*F*_IT_ * *=* *0.123	.000**

**Table A5:** migrate‐N model selection using the approximate log marginal likelihood (lmL) method. The Bézier approximation score was used to calculate the log‐equivalent Bayes factor (LBF) and select the most probable model (in bold) from among these four models. *P*_Mi_ is the model choice probability

Model	No. of parameters	Bézier lmL	LBF	*P*_Mi_
*Mustelus mustelus*				
1. Full	4	−21557.47	2086.26	0.00
2. To SEAO only	**3**	**−20514.34**	**0.00**	**1.00**
3. To SWIO only	3	−20577.89	127.10	0.00
4. Panmictic	1	−29466.69	17904.70	0.00
*Mustelus palumbes*				
1. Full	4	−17365.13	5456.48	0.00
2. To SEAO only	**3**	**−14636.89**	**0.00**	**1.00**
3. To SWIO only	3	−14797.22	320.66	0.00
4. Panmictic	1	−21372.88	13471.98	0.00
*Galeorhinus galeus*				
1. Full	4	−12243.70	15635.98	0.00
2. To SEAO only	**3**	**−4425.71**	**0.00**	**1.00**
3. To SWIO only	3	−4502.19	152.96	0.00
4. Panmictic	1	−5765.09	2678.76	0.00
*Triakis megalopterus*				
1. Full	4	−15757.39	12746.02	0.00
2. To SEAO only	**3**	**−9384.38**	**0.00**	**1.00**
3. To SWIO only	3	−9450.22	131.68	0.00
4. Panmictic	1	−12549.03	6329.30	0.00

**Table A6:** Results from migrate‐N for model 2 including parameters, the mode of the posterior distribution of the migration parameter *M* and bounds of 95% confidence intervals, the Θ and *N*_e_ *m* (product of *M* and Θ divided by 4). SEAO is the Southwast Atlantic Ocean and SWIO is the Southwest Indian Ocean basins, respectively

Species	Parameter	M mode	M 2.5%	M 97.5%	Mean
*Mustelus mustelus*	Θ_SEAO_	0.79	0.40	1.32	0.86
	Θ_SWIO_	5.87	4.94	6.88	5.91
	*M*_SWIO→SEAO_	37.95	29.00	49.70	38.63
	*N*_*e*_ *m*	7.50			
*Mustelus palumbes*	Θ_SEAO_	0.54	0.08	0.96	0.53
	Θ_SWIO_	19.66	18.56	20.00	19.32
	*M*_SWIO→SEAO_	4.25	2.00	7.80	4.81
	*N*_*e*_ *m*	0.57			
*Galeorhinus galeus*	Θ_SEAO_	0.10	0.00	1.60	0.23
	Θ_SWIO_	98.10	76.80	100.00	90.01
	*M*_SWIO→SEAO_	3.80	0.00	9.20	4.16
	*N*_*e*_ *m*	0.10			
*Triakis megalopterus*	Θ_SEAO_	1.38	0.28	15.36	4.77
	Θ_SWIO_	6.82	5.64	8.04	6.85
	*M*_SWIO→SEAO_	89.40	52.00	146.40	96.59
	*N*_*e*_ *m*	30.84			
